# Possible Reasons for Limited Effectiveness of a Skills and Drills Intervention to Improve Emergency Obstetric and Newborn Care

**DOI:** 10.9745/GHSP-D-17-00055

**Published:** 2017-03-24

**Authors:** Helen A Allott, Helen Smith, Terry Kana, Mselenge Mdegela, Sarah Bar-Zeev, Charles Ameh

**Affiliations:** a Liverpool School of Tropical Medicine, Centre for Maternal and Newborn Health, Liverpool, UK.

See related article by Varghese.

We read with interest the paper by Varghese et al.^1^ regarding the limited effectiveness of a skills and drills intervention to improve emergency obstetric and newborn care and the accompanying editorial by Ricca.^2^

Ricca discussed some possible reasons as to why the intervention had limited effect when it came to improved diagnosis and management of maternal and newborn complications, including systems weaknesses, provider motivation and behavior, and barriers to teamwork in the workplace.

We would like to draw attention to a further possible reason for the limited effectiveness in translating the training into demonstrable improvements in clinical care. While there were statistically significant improvements in both knowledge and skills, as assessed by pre- and post-intervention knowledge and skills assessments, it may be that these improvements, albeit of significance, still did not cross a threshold of the improvement necessary to make a real difference in clinical practice. It could be argued, for example, that a score of 56% in understanding how to recognize and act in an obstetric emergency is simply still not enough.

We would, therefore, suggest that prior to implementing any further such intervention, both the content and mode of delivery of the training intervention be re-explored with a view to gaining an understanding as to why it was that participants' scores did not reach a higher level. Then appropriate changes can be implemented in the training in order to achieve a greater demonstrable level of knowledge and skills improvement, which may be more likely to have an impact on clinical practice.

In our experience in the multi-country Making It Happen program,^3^ setting up skills training rooms^4^^,^^5^ and training of health care facility-based mentors and supervisors were successful approaches in bringing about change in behavior and practice after training.

As a final point, evaluating the effectiveness of training programs is the last step of an effective training program design but in order to improve the strength of the results, attention must be paid to more robust designs beyond pre- and post-training assessments. Having a matched comparison group with outcome indictors linked to the training intervention will minimize bias associated with the results. The stepped wedge research design^6^ allows all clusters to receive the intervention at various times while being part of the control group at some point. This design is better suited for when it is known that an intervention has benefits, and therefore it is unethical to withhold the intervention from other groups.
